# Transcriptomic and metabolomic profiling of chicken adipose tissue in response to insulin neutralization and fasting

**DOI:** 10.1186/1471-2164-13-441

**Published:** 2012-08-31

**Authors:** Bo Ji, Ben Ernest, Jessica R Gooding, Suchita Das, Arnold M Saxton, Jean Simon, Joelle Dupont, Sonia Métayer-Coustard, Shawn R Campagna, Brynn H Voy

**Affiliations:** 1 Department of Animal Science, University of Tennessee, Knoxville, Tennessee, USA; 2 Department of Chemistry, University of Tennessee, Knoxville, Tennessee, USA; 3 Unité de Recherches Avicoles (U83), Institut National de la Recherche Agronomique (INRA), Nouzilly, 37380, France; 4 Unité de Physiologie de la Reproduction et des Comportements (UMR85), Institut National de la Recherche Agronomique (INRA), Nouzilly, 37380, France; 5 201E McCord Hall, Morgan Circle Dr. Knoxville, Tennessee, 2640, USA

**Keywords:** Microarray, Chicken adipose tissue, Fasting, Insulin neutralization, Fatty acid metabolism, Glucose metabolism, Adipogenesis

## Abstract

**Background:**

Domestic broiler chickens rapidly accumulate adipose tissue due to intensive genetic selection for rapid growth and are naturally hyperglycemic and insulin resistant, making them an attractive addition to the suite of rodent models used for studies of obesity and type 2 diabetes in humans. Furthermore, chicken adipose tissue is considered as poorly sensitive to insulin and lipolysis is under glucagon control. Excessive fat accumulation is also an economic and environmental concern for the broiler industry due to the loss of feed efficiency and excessive nitrogen wasting, as well as a negative trait for consumers who are increasingly conscious of dietary fat intake. Understanding the control of avian adipose tissue metabolism would both enhance the utility of chicken as a model organism for human obesity and insulin resistance and highlight new approaches to reduce fat deposition in commercial chickens.

**Results:**

We combined transcriptomics and metabolomics to characterize the response of chicken adipose tissue to two energy manipulations, fasting and insulin deprivation in the fed state. Sixteen to 17 day-old commercial broiler chickens (ISA915) were fed *ad libitum*, fasted for five hours, or fed but deprived of insulin by injections of anti-insulin serum. Pair-wise contrasts of expression data identified a total of 2016 genes that were differentially expressed after correction for multiple testing, with the vast majority of differences due to fasting (1780 genes). Gene Ontology and KEGG pathway analyses indicated that a short term fast impacted expression of genes in a broad selection of pathways related to metabolism, signaling and adipogenesis. The effects of insulin neutralization largely overlapped with the response to fasting, but with more modest effects on adipose tissue metabolism. Tissue metabolomics indicated unique effects of insulin on amino acid metabolism.

**Conclusions:**

Collectively, these data provide a foundation for further study into the molecular basis for adipose expansion in commercial poultry and identify potential pathways through which fat accretion may be attenuated in the future through genetic selection or management practices. They also highlight chicken as a useful model organism in which to study the dynamic relationship between food intake, metabolism, and adipose tissue biology.

## Background

The domestic chicken provides a widespread and relatively inexpensive source of dietary protein for humans. In addition to its role as a food animal, the chicken has a long history as a valuable model research organism [1]. These dual considerations led to the selection of chicken as the first agricultural animal model to be sequenced at the genome level [2]. While chickens have been used heavily for studies of developmental biology and immunology, a number of traits make them a viable model for studies of adipose biology, obesity and insulin resistance. Commercial broiler chickens, in particular, rapidly accumulate excess adipose tissue as a result of genetic selection for growth and are considered “obese” relative to leaner egg-laying or wild strains of chickens(rev. in [3]). Chickens mimic the early stage of type 2 diabetes in humans, exhibiting both hyperglycemia (up to 200 mg/dL in the fasting state) and resistance to exogenous insulin [4,5]. Like humans, but unlike rodents or pigs, chickens rely on liver rather than adipose tissue for the majority of *de novo* lipid synthesis [6-8]. Most metabolic genes are conserved with humans, and a number of the quantitative trait loci (QTLs) that have been linked to fatness in chickens contain genes implicated in human susceptibility to obesity or diabetes [9]. Chickens also represent a model for studying mechanisms of adipocyte hyperplasia during development, a process that may exacerbate adult obesity. During at least the first several weeks after hatch, chicken adipose tissue expands more through adipocyte hyperplasia than hypertrophy, and an early increase in adipocyte number is a common feature of some lines genetically selected for excess adiposity [10,11]. Finally, the egg presents opportunities to directly manipulate the developmental milieu and study the consequences on adipose metabolism via *in ovo* injection.

Relatively little is known about regulation of adipose tissue deposition and metabolism in chicken. Because of its relative importance in lipogenesis, most studies have focused on the role of liver in adipose expansion. Several genetic lines of fat and lean chickens have been developed through phenotypic selection, most of which have both elevated plasma levels of very low density lipoprotein (VLDL) and lower levels of plasma glucose, reflecting the importance of hepatic lipogenesis and glucose consumption in fat accretion. Reciprocally, phenotypic selection for low plasma glucose simultaneously selects for fatness [12]. Both chicken and mammalian adipocytes develop through a sequence of molecular triggers including activation of CCAAT-enhancer-binding protein alpha (CEBPα) and peroxisome proliferator-activated receptor gamma (PPARγ) [13]. A clear point of divergence, however, is their responsiveness to insulin. Unlike in mammals, insulin has minimal effect on glucose uptake in chicken adipose tissue [14]. In fact, an avian homolog of the insulin-sensitive glucose transporter GLUT4 has not been identified in the current chicken genome database. Insulin does, however, stimulate uptake of acetate, which is the preferred substrate for *de novo* lipogenesis in chicken adipocytes, although the magnitude of the effect is relatively modest [15]. Insulin signaling appears to proceed through tissue specific cascades in chicken metabolic tissues. In liver, insulin elicits a signaling cascade that parallels the response in mammals, including tyrosine phosphorylation of insulin receptor β-subunit (IRβ), insulin receptor substrate-1 (IRS-1) and Src homology 2 domain-containing substrate (Shc) and activation of phosphatidylinositol 3-kinase (PI3K) [16,17]. The situation in skeletal muscle is more complex. Tyrosine phosphorylation of IRβ and IRS-1 and PI3K activity are not regulated by insulin, whereas events downstream of PI3K (e.g. Akt and P70S6K activation) are accordingly sensitive [18]. We recently reported that insulin also does not elicit a classical IRβ initiated cascade in chicken adipose tissue, including the downstream steps of Akt and P70S6K activation [19]. Insulin also does not inhibit lipolysis in chicken adipose tissue; glucagon, is the primary lipolytic hormone (rev. in [20]).

In the present study we simultaneously characterized the effects of a short term (5 hours) fast or neutralization of insulin action (5 hours) on adipose tissue of young (16–17 day-old), fed commercial broiler chickens. The goals of this study were two-fold. First, we sought to identify pathways activated by feed restriction, reasoning that they may highlight potential strategies for control of fatness through either genetic selection or improved management practices. Simultaneously, we sought to understand the contribution of insulin, if any, into chicken adipose physiology. No experimental model of diabetes exist in chicken: total pancreatectomies are not achievable, and alloxan and streptozotocin are inefficient at destroying pancreatic chicken beta-cells (rev. in [5]). The two treatments were compared to distinguish potential insulin-specific changes from those that could be mimicked by fasting through changes in nutrient availability. Both treatments were shown previously to elicit significant alterations in several plasma metabolic and endocrine parameters [18]; in the studies reported herein, samples of abdominal adipose tissue were issued from the same experiment. Tissue metabolomics was combined with microarrays to bridge the gap between gene expression, metabolic and physiological responses, and to identify the composite effects of both fasting and insulin deprivation on chicken adipose tissue.

## Results

**Figure 1:**
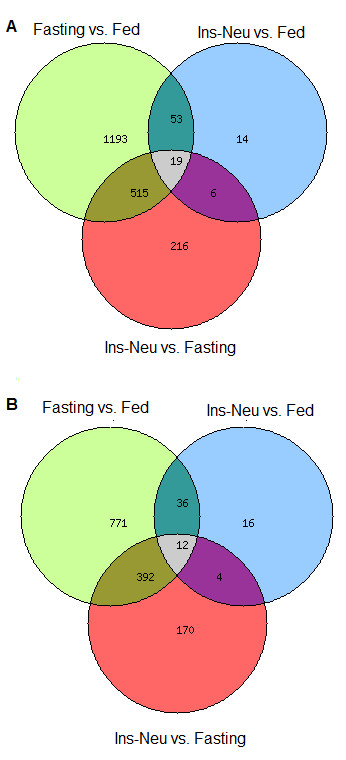
**Venn diagram of overlapping and unique effects of fasting and insulin neutralization on gene expression.** (**A**) A total of 2016 unique genes were differentially expressed (FDR adjusted p-value <0.05) between one or more pairwise treatment comparisons; (**B**) A total of 1401 genes with absolute fold change ≥1.5 among the differentially expressed genes.

Expression levels of a total of 2016 genes were significantly altered by fasting and/or insulin neutralization when compared to fed controls based on an FDR adjusted p-value < 0.05 ( Additional file 1; Figure 1A). Sixty-nine percent of these genes showed a fold-change ≥ |1.5| (Figure 1B). The majority of changes in expression were attributable to fasting, with 917 up-regulated and 863 down-regulated genes in fasted vs. fed adipose tissue. Insulin neutralization altered expression of 92 genes, 72 of which were also differentially expressed with fasting (Figure 1A). All genes that were affected by both treatments changed in the same direction (i.e., up- or down-regulated in both groups). Real-time RT-PCR was employed to validate differential expression based on the microarray data. Eleven genes were selected based on fold-change or biological functions of interest (Table 1). Differential expression under fasting versus fed conditions was validated for all genes except pre-B-cell leukemia homeobox 3 (PBX3). Ten of the eleven genes were also differentially expressed in insulin neutralized compared to fed birds based on QPCR.

**Table 1:** Fold change verification of gene expression by RT-PCR

**Gene symbol**	**Gene title**	**Microarray fold change**			**Quantitative PCR fold change**		
		**Fasting vs. Fed**	**Ins-Neu vs. Fed**	**Ins-Neu vs. Fasting**	**Fasting vs. Fed**	**Ins-Neu vs. Fed**	**Ins-Neu vs. Fasting**
FBXO8	F-box protein 8	4.16***	2.09**	−1.99***	2.59***	2.76***	1.06
DUSP5	Dual specificity phosphatase 5	9.43***	1.05	−8.98***	8.13***	1.69***	−4.82***
BNIP3	BCL2/adenovirus E1B 19 kDa interacting protein 3	2.98***	1.53	−1.95**	3.45***	2.26***	−1.52*
PBX3	Pre-B-cell leukemia homeobox 3	−1.62***	1.07	1.75***	−1.22	1.83***	2.22***
IL10RB	Interleukin 10 receptor, beta	1.74***	1.02	−1.71**	1.76***	1.42*	−1.24
EGR1	Early growth response 1	2.43*	−1.58	−3.86**	2.58***	−1.36	−3.52***
NAB1	NGFI-A binding protein 1 (EGR1 binding protein 1)	2.43***	1.06	−2.29***	1.67**	1.52**	−1.10
PDK4	Pyruvate dehydrogenase kinase, isozyme 4	17.28***	7.06**	−2.45**	18.33***	4.01***	−4.57***
CTSL2	Cathepsin L2	2.09***	1.55*	−1.35*	2.96***	2.053***	−1.44
AGTR1	Angiotensin II receptor, type 1	4.15 ***	2.05*	−2.02**	3.52**	1.72*	−2.05*
SESN1	Sestrin 1	1.86**	1.125	−1.65	1.58*	1.47*	1.07

FDR p-value: * p < 0.05, ** p < 0.005, *** p < 0.0005.

**Figure 2:**
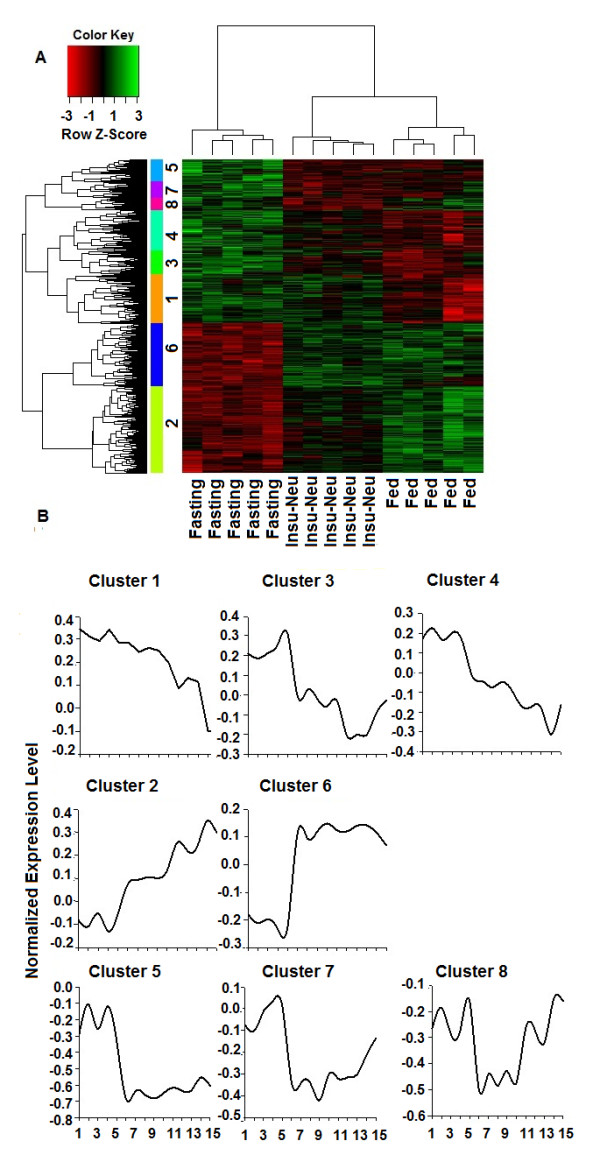
**Cluster analysis of differentially expressed genes.** The 2016 genes differentially expressed in one or both treatment groups vs. fed controls were subjected to hierarchical clustering to visualize similarities and differences between treatment groups. (**A**). Hierarchical cluster analysis of the 2016 genes (FDR adjusted p-value <0.05) that were differentially expressed between insulin-neutralized vs. fed and/or fasted vs. fed states. (**B**) Seven clusters (numbers) representing the most distinct effects of treatment were selected to further analyze expression profiles across treatments. Sample ID number on the X-axis corresponds to treatment group: sample 1–5, fasted; 6–10, insulin neutralized; 11–15,fed control. Y-axis represents relative gene expression value.

Genes that were differentially expressed in at least one pairwise comparison were clustered to visualize the similarities between groups and to determine if insulin-neutralized expression profiles were more similar to fasted or to fed status. As shown in Figure 2A, samples within each of the three experimental groups clustered together. The dendrogram also showed that the fasting group was distant from fed and insulin-neutralized groups, which were closer to each other. To further visualize relationships between treatments with regard to gene expression, distinct clusters of genes were extracted and submitted to gene set enrichment analysis to identify GO terms and pathways that were significantly overrepresented among genes contained in these clusters. Seven clusters represented four general patterns of similarities between treatments (Figure 2B). Clusters 1, 3 and 4 consisted of genes with higher expression in fasting compared to both insulin-neutralized and fed conditions, with insulin-neutralized intermediate between fasted and fed. This set of genes was significantly enriched in GO terms related to protein and lipid catabolism and to cell signaling, including regulation of the stress-sensitive NFκB cascade (Table 2). These three clusters were also enriched in members of the KEGG pathways ubiquitin mediated proteolysis, sphingolipid metabolism, PPAR signaling, fatty acid metabolism and the peroxisome. The rate-limiting genes for fatty acid oxidation (ACOX1 and CPT1A), along with fatty acid binding proteins 5 and 6, are contained in these three clusters. Clusters 5 and 7 also contained genes with higher levels in fasted vs. the other two groups, but with comparable expression levels between insulin-neutralized and fed, and thus no clear effect of insulin loss. These two clusters were significantly enriched in pathways related to signaling and metabolism, including enzyme linked receptor protein signaling pathway (p = 0.0097) and in the KEGG pathways for glycerolipid metabolism and PPAR. Genes responsible for the latter enrichment include PPARΔ, which was recently shown to increase total oxidative metabolism in white adipose tissue [21]. Clusters 2 and 6 contained genes expressed at lowest levels in fasted chickens. Genes in cluster 2 were expressed at intermediate levels in the insulin-neutralized group relative to fed and fasted. This set of genes was significantly enriched in GO annotations related to monosaccharide catabolic process and glucose metabolism, and in genes comprising the KEGG pathways for carbohydrate metabolism, TCA cycle and glycolysis (Table 2). Finally, cluster 6 consisted of genes that were also lowest in fasting but showed no clear effect of insulin loss, with similar expression in fed and insulin-neutralized groups. This set of genes was significantly enriched for the KEGG pathways steroid biosynthesis (p = 0.0043), glyoxylate and dicarboxylate metabolism (P = 0.012) and pyruvate metabolism (P = 0.033), along with a number of genes involved in lipid biosynthesis, which was the highest scoring GO category (p = 0.6). Cluster 8 was a distinct, small cluster with variable expression within group and no significant GO or KEGG annotations. 

**Table 2:** Gene ontology (GO) and KEGG annotation for representative clusters of differentially expressed genes

**Cluster**	**Annotation**	**GO term (Biological Process, level 6 or 7) or KEGG pathway name**	**FDR p-value**
1, 3 and 4 (731 genes)	GO	Positive regulation of protein metabolic process	6.0 E-3
		Negative regulation of cellular macromolecule biosynthetic process	7.3 E-3
		Triglyceride metabolic process	1.0 E-2
		Negative regulation of gene expression	1.0 E-2
		Proteolysis involved in cellular protein catabolic process	1.0 E-2
		Negative regulation of kinase activity	1.5 E-2
		Regulation of transcription, DNA-dependent	2.1 E-2
		Protein phosphorylation	3.3 E-2
		Antigen receptor-mediated signaling pathway	3.3 E-2
		Regulation of phosphate metabolic process	3.4 E-2
		Regulation of kinase activity	3.4 E-2
		Regulation of I-kappaB kinase/NF-kappaB cascade	3.5 E-2
	KEGG	Ubiquitin mediated proteolysis	1.0 E-2
		Sphingolipid metabolism	1.8 E-2
		PPAR signaling pathway	2.4 E-2
		Fatty acid metabolism	4.6 E-2
		Peroxisome	5.0 E-2
2 (557 genes)	GO	Monosaccharide catabolic process	2.5 E-2
		DNA dependent DNA replication	3.6 E-3
		Hexose metabolic process	1.1 E-2
		Glucose metabolic process	1.4 E-2
		Regulation of cell shape	1.5 E-2
		DNA replication	1.5 E-2
		Nucleoside triphosphate metabolic	2.3 E-2
	KEGG	Glycolysis / Gluconeogenesis	4.9 E-4
		Citrate cycle (TCA cycle)	8.9 E-4
6 (402 genes)	KEGG	Steroid biosynthesis	4.4 E-3
		Glyoxylate and dicarboxylate metabolism	1.2 E-2
		Pyruvate metabolism	3.3 E-2
5 and 7 (250 genes)	GO	Enzyme linked receptor protein signaling pathway	9.7 E-3
		Regulation of cellular protein metabolic process	3.1 E-2
		Negative regulation of macromolecule metabolic process	3.5 E-2
		Transmembrane receptor protein serine/threonine kinase signaling pathway	3.6 E-2
		Negative regulation of protein metabolic process	3.7 E-2
		Positive regulation of cellular metabolic process	4.4 E-2
	KEGG	PPAR signaling pathway	1.0 E-4
		Glycerolipid metabolism	1.8 E-2
		MAPK signaling pathway	4.6 E-2

Global biological responses to fasting and to insulin neutralization were further characterized using KEGG pathway matching, based on genes with statistically significant differential expression (FDR < 0.05) and absolute fold-change ≥1.5. Genes altered exclusively by fasting represented a wide range of cellular pathways, indicating significant effects of even a five hour fast on adipose function and metabolism in chicken. Fasting exerted significant effects on pathways related to carbohydrate, amino acid and lipid metabolism and synthesis. Within the categories related to lipid metabolism, fasting up-regulated expression of genes involved in fatty acid oxidation (e.g., acyl-CoA oxidase 1 (ACOX1), acetyl-CoA carboxylase beta (ACACB), carnitine palmitoyltransferase 1A (CPT1A)) and down-regulated expression of genes that control fatty acid, cholesterol and triacylglycerol synthesis (e.g., 1-acylglycerol-3-phosphate O-acyltransferase 9 (AGPAT9), ATP citrate lyase (ACLY), farnesyl diphosphate synthase (FDPS), acetyl-Coenzyme A carboxylase alpha (ACACA) and acetoacetyl-CoA synthetase (AACS)). Fasting also up-regulated expression of many genes involved in proteolysis and amino acid degradation. In addition to pathways highlighted by KEGG analysis, fasting down-regulated a number of genes (e.g., TGFβ, BMP) that mediate mesenchymal stem cell (MSC) commitment, an early step in the formation of new adipocytes ( Additional file 1). Finally, a number of phosphodiesterases were up-regulated with fasting, presumably in response to the increased plasma glucagon [18] and subsequent elevations in cyclic adenosine monophosphate (cAMP; Additional file 1). Collectively, these categories indicate that chicken adipose tissue responds to a relatively short duration (five hour) fast with sweeping changes in gene expression that suppress synthesis and storage of lipids and other macromolecules and up-regulate mobilization and metabolism of fatty acids and proteins.

Loss of insulin action also resulted in significant effects on adipose gene expression, the majority of which overlapped with the response to fasting (Table 3; Additional file 2). Several genes central to energy metabolism were affected. Diacylglycerol O-acyltransferase homolog 2 (DGAT2), which catalyzes the final and only committed step in triacylglycerol synthesis, was down-regulated (10.5-and 6.1-fold, respectively, fasted and insulin neutralized) in both treatment groups relative to the fed group. Conversely, acyl-Coenzyme A binding domain containing 5 (ACBD5) and pyruvate dehydrogenase kinase 4 (PDK4) were significantly up-regulated in both treatments relative to fed controls. ACBD5 is one of a family of long chain fatty acyl CoA trafficking proteins that play roles in both triglyceride synthesis and beta-oxidation [22]. PDK4, which was up-regulated vs. fed by ~ 17-fold with fasting and 6-fold with insulin neutralization, acts as a fuel switch by phosphorylating and inactivating pyruvate dehydrogenase, shifting metabolism from glycolysis to fatty acid oxidation [23]. Fasting and insulin neutralization also up-regulated expression of the type I angiotensin II receptor (AGTR1). Angiotensin II alters adipocyte lipid metabolism and insulin signaling [24-26], and increased AGTR1 expression in adipose tissue is associated with enhanced insulin sensitivity [27]. Finally, a number of genes regulated by both fasting and insulin neutralization function in general processes related to protein synthesis. 

**Table 3:** Shared effects of fasting and insulin-neutralization on differential gene expression

**Gene symbol**	**Gene title**	**Fold change Insu-Neu vs. Fed**	**Fold change Fasting vs. Fed**
**Up-regulated genes**			
PDK4	pyruvate dehydrogenase kinase, isozyme 4	7.06	17.28
AOX1	aldehyde oxidase 1	2.94	6.66
PLEKHH2	pleckstrin homology domain containing, family H (with MyTH4 domain) member 2	2.41	2.77
FBXO8	F-box protein 8	2.09	4.15
AGTR1	angiotensin II receptor, type 1	2.04	4.15
PCMTD1	protein-L-isoaspartate (D-aspartate) O-methyltransferase domain containing 1	1.99	2.25
PSME4	proteasome (prosome, macropain) activator subunit 4	1.97	3.86
ICA1	islet cell autoantigen 1, 69 kDa	1.85	2.8
IP6K2	inositol hexakisphosphate kinase 2	1.77	2.75
UHRF2	ubiquitin-like, containing PHD and RING finger domains, 2	1.72	1.89
ACBD5	acyl-Coenzyme A binding domain containing 5	1.72	2.51
LOC417776	similar to hypothetical protein	1.62	1.84
CTSL2	cathepsin L2	1.54	2.09
ZNF217	zinc finger protein 217	1.54	2.6
IFNAR1	Interferon (alpha, beta and omega) receptor 1	1.52	3.47
**Down-regulated genes**			
DGAT2	diacylglycerol O-acyltransferase homolog 2 (mouse)	6.1	10.5
EEPD1	endonuclease/exonuclease/phosphatase family domain containing 1	2.4	2.17
ANKRD9	ankyrin repeat domain 9	2.19	1.98
DLST	dihydrolipoamide S-succinyltransferase (E2 component of 2-oxo-glutarate complex	1.95	1.76
PTP4A3	protein tyrosine phosphatase type IVA, member 3	1.8	2.3
HSPA5	heat shock 70 kDa protein 5 (glucose-regulated protein, 78 kDa)	1.76	1.74
NOLA2	nucleolar protein family A, member 2 (H/ACA small nucleolar RNPs)	1.73	1.57
MST1R	macrophage stimulating 1 receptor (c-met-related tyrosine kinase)	1.71	2.2
GRAMD2	GRAM domain containing 2	1.69	2.37
DHDDS	dehydrodolichyl diphosphate synthase	1.63	1.58
DYNLL2	dynein, light chain, LC8-type 2	1.6	2.52
CDT1	chromatin licensing and DNA replication factor 1	1.57	1.51
FAHD1	fumarylacetoacetate hydrolase domain containing 1	1.56	1.61
DOT1L	DOT1-like, histone H3 methyltransferase (S. cerevisiae)	1.56	1.82
BTBD11	BTB (POZ) domain containing 11	1.55	1.71

FDR p-value <0.05.

A total of thirteen genes (four up-regulated and nine down-regulated) were differentially expressed only with insulin neutralization (Table 4). The most interesting of these responses were upregulation of GCG, which encodes preproglucagon (fold change = 2.91), in parallel with downregulation of the glucagon receptor (LOC425670, fold change = −2.77). Other genes uniquely affected by insulin have less clear relevance to adipose biology according to current knowledge.

**Table 4:** Unique effects of insulin neutralization on differential gene expression

**Up-regulated genes**			**Down-regulated genes**		
**Gene symbol**	**Gene title**	**Fold change**	**Gene symbol**	**Gene title**	**Fold change**
GCG	Glucagon	2.91	LOC425670	glucagon receptor precursor	2.77
TCP11L2	t-complex 11 (mouse)-like 2	2.08	BAK1	BCL2-antagonist/killer 1	1.83
LOC416916	hypothetical LOC416916	1.98	CCT3	chaperonin containing TCP1, subunit 3 (gamma)	1.62
MAGI1	membrane associated guanylate kinase, WW and PDZ domain containing 1	1.84	SETD7	SET domain containing (lysine methyltransferase) 7	1.62
SEPT10	septin 10	1.79	ST13	suppression of tumorigenicity 13 (colon carcinoma) (Hsp70 interacting protein)	1.62
LSM14A	LSM14A, SCD6 homolog A (S. cerevisiae); similar to LSM14 homolog A (SCD6, S. cerevisiae)	1.71	AHSA1	AHA1, activator of heat shock 90 kDa protein ATPase homolog 1 (yeast)	1.62
			TOE1	target of EGR1, member 1 (nuclear)	1.59
			AZIN1	antizyme inhibitor 1	1.56

FDR p-value <0.05.

**Figure 3:**
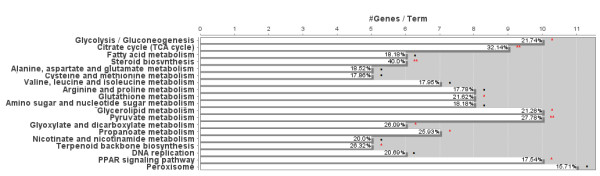
**KEGG pathway analysis of genes differentially expressed in fasting vs. fed.** Genes differentially expressed in fasting vs. fed were matched to KEGG pathway membership using ClueGO. The percentage and #genes/term indicates the percentage and number of the genes in the pathway that are contained in the set of genes altered by fasting. · p < 0.1 * p < 0.05 **, p < 0.01, based on Benjamini-corrected p-value.

**Figure 4:**
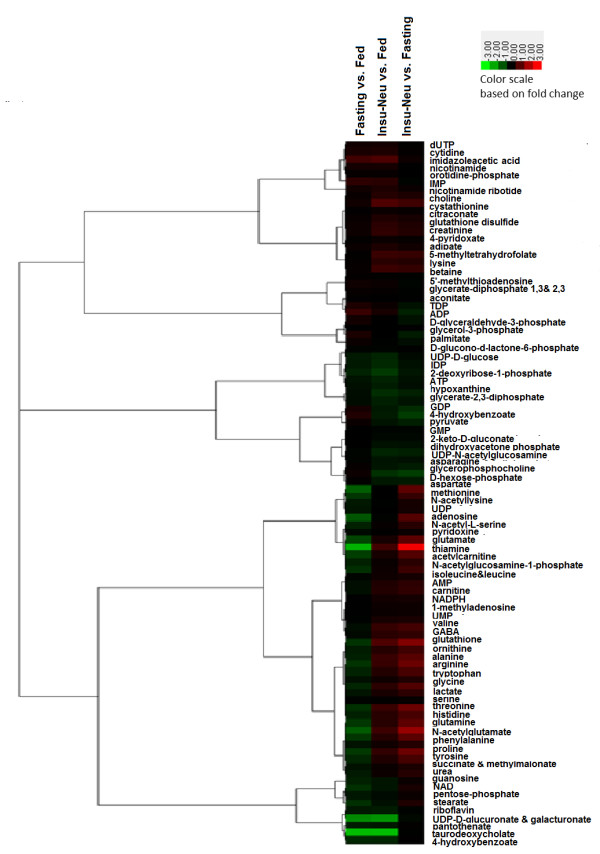
**Heat map of metabolites.** The median value of each metabolite in each treatment group was used to calculate fold-change of fasted vs. fed, insulin-neutralized (insneut) vs. fed, and insulin-neutralized vs. fasted, and then values were subjected to hierarchical clustering.

Tissue metabolomic analysis was used to identify the metabolic intermediates that were altered by fasting and insulin neutralization. A total of 92 metabolites were detected based on signal-to-noise ratios ( Additional file 3). It is worth noting that glucose-6-phosphate content was similar in fasted or “diabetic” *vs.* fed status, despite a large range of plasma glucose levels (232–747 mg/100 ml). A total of 12 metabolites were significantly different between treatment groups based on p < 0.05 and an additional five were suggestive of significance (p < 0.10; Table 5). Tissue levels of amino acids were consistently lower in fasted vs. fed tissue, with statistically significant reductions in asparagine and glutamine (p < 0.01). Presumably, these effects were due to a change in the balance of protein synthesis/proteolysis and to the catabolism of carbon skeletons for energy in response to energy restriction, which is consistent with up-regulated expression of genes involved in amino acid catabolism (Figure 3). They may also reflect a decrease in plasma amino acid supply as suggested by the decrease in total plasma amino acid levels (evaluated by the content in total α-NH_2_-non-protein nitrogen (αNH_2_NPN), i.e., mostly total amino acids), as compared to fed controls [18]. In contrast to fasting, tissue amino acid levels tended to be increased in insulin-neutralized vs. fed, although only glutamine showed a statistically significant response. Comparison of insulin-neutralized vs. fasted chickens highlights the divergent effects of treatments on amino acids (Figure 4). Alanine, arginine, asparagine, glutamine, histidine, proline, serine, threonine and tyrosine levels were all significantly higher in insulin-neutralized vs. fasted, with differences ranging from 1.7- to 3.4-fold. Two metabolites related to glucose metabolism, D-glucono-1,5-lactone-6-phosphate and glycerol-3-phosphate, were lower in both fasted and insulin-neutralized treatments vs. fed, with the latter comparison nearing statistical significance (p < 0.1). D-glucono-1,5-lactone-6-phosphate is a product of glucose-6-phosphate dehydrogenase (G6PDH), an enzyme that, in mammals is insulin-sensitive and rate-limiting for pentose phosphate pathway activity and production of cellular NADPH, an important cofactor for lipid metabolism [28]. However, pentose phosphate pathway activity is intrinsically low in chicken and is not stimulated when lipogenesis is high; the production of cellular NADPH is more closely related to malic enzyme activity [29]. Glycerol-3-phosphate is a product of both glucose and pyruvate metabolism and is used in triacylglycerol synthesis. Lower levels with both treatments may reflect glycerol demand for fatty acid reesterification in light of the apparent increase in lipolysis in both treatment groups [18]. 

**Table 5:** Fold change of fasting and insulin neutralization on adipose tissue metabolites

**Metabolite**	**Fasting/ Fed**	**Ins-Neu/ Fed**	**Ins-Neu/ Fasting**
adenosine	0.57	1.21	2.12*
alanine	0.66	1.57	2.38***
arginine	0.75	1.34	1.78**
asparagine	0.47***	1.00	2.15***
D-glucono-1,5-lactone-6-phosphate	0.86	0.75*	0.87
glutamine	0.48***	1.64*	3.43****
glycerol-3-phosphate	0.82	0.72*	0.87
histidine	0.64	1.38	2.17***
hypoxanthine	1.31	0.80	0.61**
N-acetyl-glutamate	0.64	1.34	2.09*
N-acetyl-L-serine	0.23**	1.70	7.30***
ornithine	0.77	1.59	2.05*
proline	0.63	1.50	2.39***
serine	0.67	1.61	2.39***
threonine	0.77	1.38	1.80**
tyrosine	0.74	1.30	1.76**
a hexose-phosphate	1.05	1.32**	1.26*

* p < 0.10, ** p < 0.05, *** p < 0.01, **** p < 0.001.

Correlated patterns of gene expression and metabolite abundance were extracted using hierarchical clustering to interconnect treatment effects on transcripts and metabolites. Clusters 2 and 3 contained genes and metabolites with lower abundance in fasted vs. fed or insulin neutralized tissue. The two clusters differed with respect to the insulin neutralized group: cluster 3 contained analytes at intermediate levels between fasted and fed, while cluster 2 contained those at levels comparable to or greater than fed. Twelve of the 17 metabolites with statistically suggestive or significant effects of treatment, including all of the amino acids and amino acid derivatives ( Additional file 4), were present in cluster 2 along with a set of genes that included the p85α regulatory subunit of PI3 kinase (PIK3R1), as well as ME, malonyl CoA decarboxylase and ELOVL6. Cluster 3 contained several metabolites including both NAD + and NADPH and was significantly enriched in GO annotations related to carbohydrate metabolism and in the KEGG pathways TCA cycle, glycolysis/gluconeogenesis, pyruvate metabolism and steroid biosynthesis (p < 0.05; Additional file 4). Clusters 7 and 8 consisted of genes and metabolites with higher levels in fasted than in the other two treatment groups. These clusters were significantly enriched in GO categories PPAR signaling and negative regulation of cellular biosynthesis and also contained citrate and pyruvate (p < 0.05; Additional file 4).

## Discussion

Despite roles as both a domestic food animal of worldwide economic importance and a widely used model organism with relevance for human obesity and insulin resistance, few studies have examined regulation of gene expression in chicken adipose tissue. To our knowledge, no studies of nutritional regulation of chicken adipose tissue at the genomic level have been reported in the published literature. Likewise, although insulin is the most well-defined hormonal mediator of metabolism in mammalian adipose tissue, its role in chicken remains to be clarified. Therefore the current study addressed two objectives: 1) characterize the transcriptomic and metabolomic response to energy manipulation as a step toward enhanced understanding of adipose biology in chicken; and 2) identify the effects of insulin on chicken adipose tissue by including a group of birds in which insulin action was blocked by immunoneutralization with an anti-insulin antibody. We sought to both identify potential new targets for genetic selection or management strategies to reduce fat accumulation in commercial broilers and to further develop chicken as a model organism for studies of human obesity.

Although intrinsic lipogenic activity is low in chicken adipose tissue, genes involved in fatty acid synthesis and storage were suppressed and those in fatty acid mobilization and oxidation were up-regulated by fasting. The 40 down-regulated genes with fold changes greater than three were significantly enriched for the GO annotation lipid biosynthetic process (FDR <0.05), including genes that control triglyceride synthesis (DGAT2 and AGPAT9) and fatty acid synthesis (ACACA, ACLY and ME), elongation (ELOVL6), and desaturation (FADS1). AGPAT9 and DGAT2 catalyze the initial and final steps, respectively, of *de novo* triglyceride synthesis. ACLY is the main enzyme for synthesis of cytosolic acetyl-CoA, which is carboxylated to malonyl-CoA by ACACA, the rate-limiting step in fatty acid synthesis. Reducing equivalents for the conversion of malonyl-CoA to palmitate are supplied by malic enzyme (ME). ELOVL6 catalyzes elongation of palmitate to stearate and appears to play a key role in insulin sensitivity [30,31]. Finally, FADS1 is rate-limiting for polyunsaturated fatty acids (PUFA) biosynthesis and was recently implicated in control of fasting glucose homeostasis in humans [32]. Genes altered by fasting in adipose tissue in this study overlapped with those shown to be differentially expressed in chicken liver after 16 or 48 hours of fasting, including ACLY, ACOX1, BCAT1 and PDK4 [33]. These authors used a different array platform than ours, which precludes precise quantitative comparisons. However, among the genes changed in both studies, the fold changes observed in adipose tissue were consistently greater than those in liver, despite the longer duration of fasting in that study. For example, PDK4 expression was up-regulated ~ 18-fold by a five hour fast in adipose tissue, but only ~ 1.5-fold after a 16 hour fast in liver. While differences in sensitivity between the two array platforms must be kept in mind, these data suggest that adipose tissue metabolism in chicken is at least as sensitive to energy status as hepatic metabolism. Our results indicate that both fatty acid synthesis and storage are dynamically regulated by energy status in chicken adipose tissue, despite its modest (~ 15%) contribution to the amount of stored fatty acids.

Both fasted and insulin-neutralized birds exhibited significant increases in plasma glucagon. Parallel elevations in plasma NEFA suggested that this resulted in significant lipolysis of stored triacylglycerol in both treatment groups. During fasting, a considerable percentage of the liberated fatty acids are re-esterified in adipocytes, and only a small fraction traditionally have been thought to be oxidized in the mitochondria of adipocytes through beta oxidation [34]. However, recent studies in mice and in human adipose tissue demonstrate that in some conditions fatty acid oxidation in white adipose tissue is considerable and may be an important determinant of obesity [35-37]. Consistent with this concept, we found significant increases in a number of key enzymes that mediate mobilization of fatty acids and their oxidation, including the rate-limiting enzymes in both mitochondrial and peroxisomal fatty acid oxidation (CPT1A and ACOX1, respectively). We measured tissue levels of beta-hydroxybutyrate, a ketone product of beta oxidation, to confirm that changes in gene expression had functional consequences and found them to be significantly elevated in adipose tissue of fasted vs. fed chickens. Levels were numerically but not statistically higher in insulin-neutralized adipose tissue (data not shown). Qualitatively, fasting-induced changes in gene expression resemble those induced by the fibrate class of drugs, which activate PPARα and promote fatty acid oxidation in white adipose tissue and are used clinically to treat hyperlipidemia [35,37-40]. These data suggest that dietary activation of PPARα, for example through supplementation with fatty acids that preferentially bind and activate this member of the PPAR family [41], may be a means to attenuate fat deposition in commercial broilers. Such action may underlie the reduced abdominal fat mass reported in broilers that were fed diets rich in n-3 PUFA [42].

Both fasting and insulin neutralization elicited marked upregulation of PDK4. PDK4 is a nutrient sensing fuel switch that phosphorylates and inactivates pyruvate dehydrogenase, which shifts fuel use from glucose to fatty acids and spares glucose for the brain during periods of fasting [23,43-45]. PDK4 also enhances glycerol synthesis in white adipose tissue by shunting pyruvate into glyceroneogenesis, at least in the fed state [46]. Hepatic and skeletal muscle expression of PDK4 is increased by fatty acids, acetyl CoA, NADH and the diabetic state and decreased by insulin and pyruvate (rev. in [23,47]). Little is known about PDK4 in chicken, but a recent study suggests it acts as a glycogen sensor in muscle and thus plays comparable roles to those in mammals [48]. In mouse white adipose tissue, PDK4 expression was shown to be induced by activation of p38MAPK [49], which we found to be significantly up-regulated with fasting and, to a lesser extent, with insulin neutralization [19]. Although PDK4 was up-regulated in both treatment groups, and both groups showed evidence of increased lipolysis [18], only fasted chickens presented a gene expression signature and tissue beta-hydroxybutyrate levels that were clearly indicative of fatty acid oxidation. Although we did not measure malonyl-CoA levels, we predict that they were reduced with fasting, but not insulin neutralization, based on reduced expression of ACACA. Malonyl-CoA allosterically binds and inhibits CPT1A, minimizing fatty acid transport and subsequent oxidation in mitochondria [50]. With insulin neutralization, increased PDK4 may thus be more aligned with the demand for glycerol needed to re-esterify fatty acids liberated by lipolysis [46]. Additional experiments are needed to confirm that manipulation of PDK4 alters fatty acid oxidation in chicken adipose tissue and to delineate its relative contributions to fatty acid oxidation and glyceroneogenesis under varying metabolic states. If manipulation of PDK4 does alter fatty acid oxidation, our results highlight this pathway as a potential target for reducing fatness, which has relevance for both poultry and humans.

Microarray data indicate that the effects of fasting in chicken adipose tissue extend beyond metabolism. GO analysis highlighted pathways such as cell cycle and cytokine-cytokine receptor interaction that are most likely related to changes in the stromal vascular fraction, which contains proliferating preadipocytes and cells of the immune system. In particular, a number of genes that regulate multiple steps in adipogenesis were significantly altered by fasting. Chickens rapidly accumulate abdominal fat after hatch, and until approximately 7 weeks of age this is due more to formation of new adipocytes than to adipocyte hypertrophy [11]. Adipocytes arise from mesenchymal stem cells in a two stage process of lineage commitment to an adipocyte fate, followed by differentiation of fibroblast-like preadipocytes into mature fat-storing cells [51]. Members of both the Wnt (MSC lineage commitment) and TGFβ/BMP (MSC lineage commitment and preadipocyte competence) signaling pathways were significantly regulated by fasting. Fasting down-regulated expression of CEBPα and PPARγ, two transcription factors that orchestrate the cascade of gene expression changes that lead to terminal adipocyte differentiation [51]. Expression of other adipogenic mediators including fibroblast growth factor 2 (FGF2), fibroblast growth factor receptor 1 (FGFR1) [52], and nuclear receptor corepressor 1 (NCOR1) [53] were also significantly regulated by fasting. Collectively, these changes suggest that adipocyte number in chickens is dynamically tied to energy status, at least in young chicks (such as those used herein) that are rapidly forming new adipocytes. An elegant study by Arner *et al*. concluded that adipocyte number in humans is a major determinant of adult fat mass and is determined during early childhood [54]. Less is known about this process in humans due to the limitations of sampling adipose tissue, particularly during development and from different abdominal depots. In light of what appears to be sensitive regulation of adipogenesis by nutritional state, chickens may thus be particularly valuable models in which to elucidate mechanisms of adipocyte hyperplasia during development that would inform the study of human obesity.

It is worth noting that, despite the uncertainty about insulin signaling in chicken adipose tissue, fasting altered the expression of several messengers encoding elements of the insulin signaling cascade. Expression of PIK3CB, which encodes the catalytic p110 subunit of PI3K, was up-regulated with fasting, while PIK3R1, which encodes the regulatory p85 subunit, was down-regulated. Such regulation could maintain some insulin signals despite a fall in plasma insulin level. CBLB and CRK, which mediate insulin signals that are associated with lipid rafts [55], were also up-regulated with fasting. In mammals, this pathway stimulates glucose uptake independently of PI3K activation, which may shed light on the apparent refractoriness of PI3K activity to insulin that was described in chicken skeletal muscle [18]. Therefore, the potential effects of insulin on lipid storage and energy utilization appear to be defended in the fasting state, when insulin levels fall, by enhanced insulin sensitivity at the post-receptor level. Additional studies are needed to confirm this effect and to further explore the potential of PI3K-independent effects of insulin on glucose utilization in chicken adipose tissue.

Insulin is not considered to be a key regulator of glucose metabolism in chicken adipose tissue, although it does induce glucose disposal in chicken liver and muscle [14]. It is therefore not surprising that the majority of genes significantly altered by both insulin neutralization and fasting are not related to glucose metabolism and lipid synthesis. The main exception is DGAT2, which catalyzes the final step in esterification of fatty acids into triglycerides. In fact, DGAT2 showed the most extreme down-regulation (6.1- and 10.5-fold, insulin-neutralized and fasted, respectively) in each treatment group, which is surprising considering that other genes related to lipogenesis were only regulated by fasting. Suppression of DGAT2 expression may be due to feedback by lipolysis, which appeared to be increased in both treatment groups based on plasma NEFA levels. In general, our data indicate that insulin deprivation altered fatty acid and glucose metabolism in a manner comparable to fasting but to a lesser extent, such that most genes involved in these pathways did not exhibit statistically significant changes in expression. For example, cluster analysis (Figure 2) revealed that some genes upregulated by fasting were also increased by insulin neutralization (clusters 1, 3 and 4); these three clusters were enriched with genes in the KEGG pathways for fatty acid metabolism and PPAR signaling, including both ACOX1 and CPT1A, among others. Similarly, among genes that were downregulated by fasting, clustering discriminated a set of genes (Figure 2, cluster 2) with a trend to also be decreased (albeit to a lesser extent than in fasting) by insulin deprivation. Interestingly, this cluster was significantly enriched in functions related to carbohydrate metabolism, suggesting that insulin does play some role in chicken adipose glucose metabolism. Comparable trends appeared in the metabolomic data. For example, stearate and palmitate (the only fatty acids measured by our MS platform) were lower (although not significantly) in both fasted and insulin neutralized compared to fed birds ( Additional file 3). While the purpose of our study design was to determine the specific effects of insulin on chicken adipose tissue, we cannot exclude the possibility that some of the overlapping changes in gene expression were secondary to systemic factors, such as hyperglucagonemia present in both treatment groups [18]. *In vitro* experiments using primary adipocytes or adipose explants will be useful to confirm specific effects of insulin on genes identified herein.

Of the 13 changes in expression that were unique to insulin neutralization, the most interesting responses were up-regulation of GCG, which encodes preproglucagon (fold change = 2.91), and down-regulation of the glucagon receptor (LOC425670, fold change = −2.77). The proglucagon system in avians is more complex than in mammals. The avian preproglucagon locus encodes two distinct precursor proteins that yield different peptides through alternative posttranslational processing: the class A transcript (PGA) yields glucagon and glucagon-like peptide-1 (GLP-1), while the class B transcript (PGB) additionally produces glucagon-like peptide-2 (GLP-2) and is more like the mammalian transcript (rev. in [56]). Adipose tissue expresses both transcripts, with PGA being slightly more abundant, and is the third highest preproglucagon expressing tissue in chicken, behind pancreas and the proventriculus [57]. We used transcript-specific QPCR to determine that only the PGB transcript was up-regulated by insulin neutralization (data not shown). Additional experiments are necessary to delineate which of the encoded peptides are up-regulated in parallel, but the coincident down-regulation of the glucagon receptor suggests a paracrine glucagon axis in chicken adipose tissue, and one that is regulated by insulin. In support of this concept, plasma glucagon (presumably derived largely from pancreas) was elevated comparably in both treatment groups [18], while GCG expression in adipose tissue was only up-regulated by insulin neutralization.

Tissue metabolomic analysis highlighted effects of insulin neutralization that were divergent from fasting and not readily apparent from microarray data. Most of the tissue amino acids that were measured were higher with insulin-neutralization but lower with fasting when each group was compared to *ad libitum* fed controls. This pattern parallels the levels of αNH_2_NPN levels in blood [18]. Low levels in fasted adipose tissue were most likely due to oxidation of the carbon skeletons for cellular energy through the tricarboxylic acid cycle (TCA) cycle and/or for glyceroneogenesis, in the absence of dietary glucose. Increased amino acid catabolism was reflected in the differential expression profiles of the fasted vs. fed comparison (Figure 3; Additional file 1). In the insulin neutralized group, however, glucose supply from food was maintained and preferentially oxidized for energy. Elevated amino acids in the insulin neutralized group may also reflect reduced utilization due to the lack of insulin’s anabolic effects, particularly on the proliferating cell population within adipose tissue. The metabolomics approach used here measured only metabolite pool sizes at the time that tissues were harvested, rather than the effect of fasting or insulin neutralization on the rates of metabolism through glycolysis and the TCA cycle. The latter would be much more informative with respect to the dynamic impact of treatment, but requires the use of isotopic labeling (e.g., by feeding ^13^C-labelled glucose) which was not performed in this study. Nonetheless, we were able to demonstrate significant effects on some metabolites that inform the parallel changes in gene expression, particularly in relation to amino acid metabolism. Combined clustering of metabolomic and gene expression together identified a set of genes correlated with many amino acid levels, including PIK3R1, ME and MCD.

## Conclusions

In summary, we determined that adipose tissue metabolism in the chicken is regulated by energy status and, to a lesser extent by insulin. Although adipose tissue is not a primary site of lipogenesis in chicken, the rate-limiting genes for fatty acid synthesis were suppressed by fasting. Likewise, fasting appeared to increase aspects of insulin sensitivity based on expression profiles, despite the view that chicken adipose tissue is relatively insensitive to insulin. Consistent with this paradigm, insulin neutralization significantly altered the expression of only a few genes related to glucose and lipid metabolism. Nonetheless, a considerable number of genes were altered by insulin neutralization, many of which thus far have unclear roles in adipose biology. Expression profiles suggest that even short term fasting alters fat storage in broilers by enhancing the oxidation of fatty acids. The initiating events that trigger upregulation of the corresponding genes are unclear, but there is considerable evidence for activation of PPARa, LXRa, and potentially other transcription factors that are activated by fatty acid ligands. Further studies are warranted to identify these triggers because of their potential impact on fat storage. Our data also suggest that broiler chicks may be an informative model organism in which to investigate dietary effects on adipose development in light of what appears to be a relationship between energy intake and adipogenesis. The results of this study thus have dual benefit for both the poultry industry and for studies of obesity in humans.

## Methods

### Animals

Male broiler chicks (ISA 915, Institut de Sélection Animale, Saint Brieuc, France) from which samples were collected for this study were hatched and raised under standard conditions, as originally described by Dupont [18] and in accordance with the guidelines for Care and Use of Agricultural Animals in Agricultural Research and Teaching. Briefly, at 16–17 days of age, chicks of similar body weights were either allowed to continue feeding (*ad libitum* fed controls), fasted for five hours, or fed but injected at 0, 2 and 4 hours with porcine anti-insulin serum (insulin neutralized). Both the fed and fasted groups received injections of normal porcine serum as a vehicle control. Abdominal adipose tissue samples were harvested and rapidly snap-frozen in liquid nitrogen, pulverized, then stored at -80°C until analysis. Adipose samples from five birds in each group were used for both microarray and metabolomic analyses.

### Gene expression

Total RNA was isolated from chicken adipose samples using the RNeasy Lipid kit and incorporating an on-column DNase treated with the RNase-free DNase Set according to the manufacturer's protocol (Qiagen.com). RNA quality and concentration were measured using the Experion System (Bio-Rad.com); only RNA passing recommended standards of quality was used for further studies. Transcriptome profiling was performed by Genome Quebec (Montreal, Canada) using the Affymetrix GeneChip Chicken Genome Array (San Diego, CA). Microarray data from this study are deposited in the Gene Expression Omnibus (GEO) under the accession number GSE35581. For real time RT-PCR validation, cDNA was synthesized using the iScript cDNA Synthesis kit (Bio-Rad.com). Commercially designed and validated primer sets (QuantiTect) and the associated SYBR Green master mix (Qiagen.com) were used to assay gene expression on a CFX96 real-time PCR detection system (Bio-Rad.com). All samples were analyzed in triplicate and normalized to ß-tubulin. Relative differences in gene expression were determined using the 2^-ΔΔCT^ method and statistical differences were tested by analysis of variance (ANOVA) [58].

### Liquid chromatography coupled with tandem mass spectrometry (LC-MS/MS)

Abdominal adipose tissue samples from five birds in each treatment group (the same five birds used for expression profiling) were extracted by placing tissue in a mortar containing liquid nitrogen and then powdering with a pestle. Portions (8–40 mg) of the powered tissue were weighed into 1.5 mL centrifuge tubes. Chilled methanol (0.5 mL at −80°C) and internal standard (5 μL of 1.7 mM benzoic acid in negative mode or 4.25 mM tris(hydroxymethyl)aminomethane in positive mode) were added to each tube. Each tube was mixed thoroughly by vortexing for two minutes, and the metabolites were extracted from the tissue for 30 min at 4°C. The tubes were then centrifuged (5 min, 4°C, 16.1 rcf) and supernatant (210 μL) was split into two autosampler vials. One of these samples was immediately placed on the LC-MS/MS for analysis, while the other was stored at −80°C for analysis in the opposite polarity ion mode on the following day.

Samples were placed in an autosampler tray chilled to 4°C, and 10 μL of each was injected onto an LC column for analysis. The chromatography method for positive ion mode was reported previously by Bajad and coworkers [59], with one exception that the column was cooled to 10°C. The chromatography method for negative ion mode was performed as reported by Waters and coworkers [60], except the gradient was allowed to run 50 min instead of 45 min to allow more thorough equilibration of the column. The eluent was introduced directly into the MS via an electrospray ionization (ESI) source fitted to a Finnigan TSQ Quantum Discovery Max triple quadrupole MS (Thermo Electron, Waltham, MA) through a 0.1 mm internal diameter fused silica capillary. The spray voltage was 4500 V in positive mode or 3000 V in negative mode. The sheath gas (nitrogen) was set to 40 psi, and the capillary temperature was set to 290°C. The collision cell gas (argon) was set to a pressure of 1.5 mTorr. Samples were analyzed using selected reaction monitoring (SRM) mode with a scan width of 1 *m/z* and a scan time of 0.05 s. The SRM parameters for most metabolites have been published previously [59]. This method was used to scan for almost 300 metabolites. Xcalibur software (Thermo Scientific, Waltham, MA) was used to manually assess the elution time of the correct LC spectral peak for each metabolite-specific SRM. The Quan Browser utility in Xcalibur was then used to integrate the LC spectral peak area (in ion counts) for each detected compound, and these data were exported to a Microsoft Excel spreadsheet for further processing.

### Statistical analysis

Statistical analysis of the microarray data was performed using R 2.9.0 and routines contained in Bioconductor (bioconductor.org). GC robust multi-array average (GCRMA) was used to normalize and scale the raw data from CEL files. The normalized data were filtered for low expression by removing any probes with normalized expression less than 3 in at least 5 arrays. Statistical significance of gene expression differences were analyzed by one-way ANOVA and empirical bayes using the limma package. Differential expression was defined based on false discovery rate (FDR) adjusted p-value <0.05. False discovery rate for differential expression and for GO and KEGG enrichment testing was controlled using the Benjamini-Hochberg method [61]. Venn diagrams of differentially expressed genes were plotted to visualize the number of differentially expressed genes for each treatment comparison and their intersections. Hierarchical clustering of significant genes was performed using the hclust function and a hierarchical clustering heatmap was created using heatmap.2 in the gplots package. Hierarchical clustering also was used to identify correlated patterns of gene expression and metabolites. The Database for Annotation, Visualization and Integrated Discovery (DAVID, version 6.7) and ClueGO, a Cytoscape plug-in, were used for Gene Ontology (GO) at level 6 and 7 and KEGG analysis of differentially expressed genes [62,63]. Statistical analysis of metabolomic data was performed using an analysis tool that we developed specifically for metabolomic data analyses (Ernest et al., manuscript in review). The script (metabR), written in the language R, uses linear mixed-effect modeling to normalize metabolomics data containing both fixed- and random-effect confounding variables. The script averages any replicate measurements (statistical sampling) made on experimental units and performs ANOVA to test for statistical differences between experimental groups.

## Abbreviations

VLDL: Very low density lipoprotein; CEBPα :CCAAT: Enhancer-binding protein alpha; PPAR: Peroxisome proliferator-activated receptor; IRβ: Insulin receptor β-subunit; IRS-1: Insulin receptor substrate-1; Shc: Src homology 2 domain-containing substrate; PI3K: Phosphatidylinositol 3-kinase; QPCR: Quantitative PCR; FDR: False discovery rate; GO: Gene ontology; PDGF: Platelet derived growth factor; MSC: Mesenchymal stem cell; Camp: Cyclic adenosine monophosphate; DGAT2: Diacylglycerol O-acyltransferase homolog 2; PDK4: Dehydrogenase kinase 4; ACBD5: Acyl-Coenzyme A binding domain containing 5; AGTR1: Type I angiotensin II receptor; PUFA: Polyunsaturated fatty acids; NEFA: Non-esterified fatty acid; ATGL: Adipose triglyceride lipase; PD: Pyruvate dehydrogenase; LXR: Liver X receptor; GCG: Preproglucagon; PGB: Preproglucagon class B transcript; BCAAs: Branched chain amino acids.

## Competing interests

The authors declare that they have no competing interests.

## Authors’ contributions

BJ performed the experimental assays, analyzed genomic data and drafted the manuscript. BE, JG and SC generated and analyzed metabolomics data. SD provided technical support and AM provided statistical support. JS, SM-C and JD performed the original in vivo study and provided tissue samples. BV designed and supervised the study and personnel and edited the manuscript. All authors read and approved the final manuscript.

## Supplementary Material

Additional file 1 **Differentially expressed genes for fasted vs. fed chickens with fold change > |1.5|.** Probe ID, gene symbol, gene title and fold change are provided. Click here for file

Additional file 2 **Differentially expressed genes for insulin neutralized vs. fed chickens.** Probe ID, gene symbol, gene title and fold change are provided. Click here for file

Additional file 3 **Metabolite levels across the three pairwise comparisons of treatments.** Metabolite, fold-change and p-value are provided. Click here for file

Additional file 4 **Metabolite-gene clusters.** Cluster, metabolite,gene Ontology and KEGG annotation and benjamini corrected pvalue are provided for clusters that contained both metabolites and genes. Click here for file

## References

[B1] Burt DW The chicken genome Genome Dyn 2006 2 123 137 18753775 10.1159/000095100

[B2] Hillier LW Miller W Birney E Warren W Hardison RC Ponting CP Bork P Burt DW Groenen MAM Delany ME Dodgson JB Chinwalla AT Cliften PF Clifton SW Delehaunty KD Fronick C Fulton RS Graves TA Sequence and comparative analysis of the chicken genome provide unique perspectives on vertebrate evolution Nature 2004 432 7018 695 716 10.1038/nature03154 15592404

[B3] RENEMA RA RUSTADME ROBINSON FE Implications of changes to commercial broiler and broiler breeder body weight targets over the past 30 years Worlds Poult Sci J 2007 63 03 457 472

[B4] Akiba Y Chida Y Takahashi T Ohtomo Y Sato K Takahashi K Persistent hypoglycemia induced by continuous insulin infusion in broiler chickens Br Poult Sci 1999 40 5 701 705 10.1080/00071669987124 10670686

[B5] Simon J Chicken as a useful species for the comprehension of insulin action Crit Rev Poultry Biol 1989 2 121 148

[B6] Shrago E Spennetta T The carbon pathway for lipogenesis in isolated adipocytes from rat, guinea pig, and human adipose tissue Am J Clin Nutr 1976 29 5 540 545 1266795 10.1093/ajcn/29.5.540

[B7] O'Hea EK Leveille GA Lipogenesis in isolated adipose tissue of the domestic chick (Gallus domesticus) Comp Biochem Physiol 1968 26 1 111 120 10.1016/0010-406X(68)90317-4 5758294

[B8] Leveille GA Romsos DR Yeh Y-Y O'Hea EK Lipid biosynthesis in the chick. A consideration of site of synthesis, influence of diet and possible regulatory mechanisms Poult Sci 1975 54 4 1075 1093 10.3382/ps.0541075 240159

[B9] Nadaf J Pitel F Gilbert H Duclos MJ Vignoles F Beaumont C Vignal A Porter TE Cogburn LA Aggrey SE QTL for several metabolic traits map to loci controlling growth and body composition in an F2 intercross between high- and low-growth chicken lines Physiol Genomics 2009 38 3 241 249 10.1152/physiolgenomics.90384.2008 19531576

[B10] Griffin HD Guo K Windsor D Butterwith SC Adipose tissue lipogenesis and fat deposition in leaner broiler chickens J Nutr 1992 122 2 363 368 1732477 10.1093/jn/122.2.363

[B11] Butterwith SC Regulators of adipocyte precursor cells Poult Sci 1997 76 1 118 123 9037698 10.1093/ps/76.1.118

[B12] Leclercq B Simon J Ricard FH Effects of selection for high and low plasma glucose concentration in chickens Br Poult Sci 1987 28 4 557 565 10.1080/00071668708416991 3446326

[B13] Wang Y Mu Y Li H Ding N Wang Q Wang S Wang N Peroxisome proliferator-activated receptor-{gamma} gene: a key regulator of adipocyte differentiation in chickens Poult Sci 2008 87 2 226 232 10.3382/ps.2007-00329 18212364

[B14] Tokushima Y Takahashi K Sato K Akiba Y Glucose uptake in vivo in skeletal muscles of insulin-injected chicks Comp Biochem Physiol B Biochem Mol Biol 2005 141 1 43 48 10.1016/j.cbpc.2005.01.008 15820133

[B15] Montes R Vives F Osorio C Gomez-Capilla JA Effect of insulin on acetate metabolism in chicken adipocytes Horm Metab Res 1981 13 12 678,681 6797910 10.1055/s-2007-1019374

[B16] Dupont J Tesseraud S Derouet M Collin A Rideau N Crochet S Godet E Cailleau-Audouin E Metayer-Coustard S Duclos MJ Insulin immuno-neutralization in chicken: effects on insulin signaling and gene expression in liver and muscle J Endocrinol 2008 197 3 531 542 10.1677/JOE-08-0055 18492818

[B17] Dupont J Derouet M Simon J Taouis M Nutritional state regulates insulin receptor and IRS-1 phosphorylation and expression in chicken Am J Physiol - Endocrinol Metab 1998 274 2 E309 E316 10.1152/ajpendo.1998.274.2.E309 9486163

[B18] Dupont J Tesseraud S Derouet M Collin A Rideau N Crochet S Godet E Cailleau-Audouin E Métayer-Coustard S Duclos MJ Insulin immuno-neutralization in chicken: effects on insulin signaling and gene expression in liver and muscle J Endocrinol 2008 197 3 531 542 10.1677/JOE-08-0055 18492818

[B19] Dupont J Metayer-Coustard S Ji B Rame C Gespach C Voy B Simon J Characterization of major elements of insulin signaling cascade in chicken adipose tissue: apparent insulin refractoriness Gen Comp Endocrinol 2012 176 1 86 93 10.1016/j.ygcen.2011.12.030 22233773

[B20] Scanes C Perspectives on the endocrinology of poultry growth and metabolism Gen Comp Endocrinol 2009 163 1–2 24 32 19393657 10.1016/j.ygcen.2009.04.013

[B21] Roberts LD Murray AJ Menassa D Ashmore T Nicholls AW Griffin JL The contrasting roles of PPARdelta and PPARgamma in regulating the metabolic switch between oxidation and storage of fats in white adipose tissue Genome Biol 2011 12 8 R75 10.1186/gb-2011-12-8-r75 21843327 PMC3245615

[B22] Mandrup S Færgeman NJ Knudsen J Duttaroy AKaS F Structure, function and phylogeny of acyl-CoA binding protein Cellular proteins and their fatty acids in health and disease 2004 Wiley-VCH gmbH & Co, Kaga, Weinheim 151 172

[B23] Sugden MC Holness MJ Recent advances in mechanisms regulating glucose oxidation at the level of the pyruvate dehydrogenase complex by PDKs Am J Physiol - Endocrinol Metab 2003 284 5 E855 E862 12676647 10.1152/ajpendo.00526.2002

[B24] Kalupahana NS Massiera F Quignard-Boulange A Ailhaud G Voy BH Wasserman DH Moustaid-Moussa N Overproduction of angiotensinogen from adipose tissue induces adipose inflammation, glucose intolerance, and insulin resistance Obesity 2012 20 1 48 56 10.1038/oby.2011.299 21979391 PMC4465436

[B25] Suyeon Kim MS-B Annie Q-B Florence M Michele T Gerard A Jung Han K Naima M-M Voy Brynn H The adipose renin-angiotensin system modulates systemic markers of insulin sensitivity and activates the intrarenal renin-angiotensin system J Biomed Biotechnol 2006 2006 27012 2006 10.1155/JBB/2006/27012 PMC1698261 17489015

[B26] Kim S Urs S Massiera F Wortmann P Joshi R Heo YR Andersen B Kobayashi H Teboul M Ailhaud G Effects of high-Fat diet, angiotensinogen (agt) gene inactivation, and targeted expression to adipose tissue on lipid metabolism and renal gene expression Horm Metab Res 2002 34 11/12 721,725 12660889 10.1055/s-2002-38263

[B27] Elbein SC Kern PA Rasouli N Yao-Borengasser A Sharma NK Das SK Global gene expression profiles of subcutaneous adipose and muscle from glucose-tolerant, insulin-sensitive, and insulin-resistant individuals matched for BMI Diabetes 2011 60 3 1019 1029 10.2337/db10-1270 21266331 PMC3046820

[B28] Gupte SA Targeting the pentose phosphate pathway in syndrome X-related cardiovascular complications Drug Dev Res 2010 71 3 161 167 20711518 10.1002/ddr.20359 PMC2918918

[B29] Goodridge AG Citrate-cleavage enzyme, 'malic' enzyme and certain dehydrogenases in embryonic and growing chicks Biochem J 1968 108 4 663 666 5667279 10.1042/bj1080663 PMC1198865

[B30] Matsuzaka T Shimano H Yahagi N Kato T Atsumi A Yamamoto T Inoue N Ishikawa M Okada S Ishigaki N Crucial role of a long-chain fatty acid elongase, Elovl6, in obesity-induced insulin resistance Nat Med 2007 13 10 1193 1202 10.1038/nm1662 17906635

[B31] Matsuzaka T Shimano H Elovl6: a new player in fatty acid metabolism and insulin sensitivity J Mol Med 2009 87 4 379 384 10.1007/s00109-009-0449-0 19259639

[B32] Dupuis J Langenberg C Prokopenko I Saxena R Soranzo N Jackson AU Wheeler E Glazer NL Bouatia-Naji N Gloyn AL New genetic loci implicated in fasting glucose homeostasis and their impact on type 2 diabetes risk Nat Genet 2010 42 2 105 116 10.1038/ng.520 20081858 PMC3018764

[B33] Desert C Duclos MJ Blavy P Lecerf F Moreews F Klopp C Aubry M Herault F Le Roy P Berri C Transcriptome profiling of the feeding-to-fasting transition in chicken liver BMC Genom 2008 9 611 10.1186/1471-2164-9-611 PMC2628918 19091074

[B34] Wang T Zang Y Ling W Corkey BE Guo W Metabolic partitioning of endogenous fatty acid in adipocytes Obes Res 2003 11 7 880 887 10.1038/oby.2003.121 12855758

[B35] Goto T Lee J-Y Teraminami A Kim Y-I Hirai S Uemura T Inoue H Takahashi N Kawada T Activation of peroxisome proliferator-activated receptor-alpha stimulates both differentiation and fatty acid oxidation in adipocytes J Lipid Res 2011 52 5 873 884 10.1194/jlr.M011320 21324916 PMC3073464

[B36] Crowe S Turpin SM Ke F Kemp BE Watt MJ Metabolic remodeling in adipocytes promotes ciliary neurotrophic factor-mediated fat loss in obesity Endocrinology 2008 149 5 2546 2556 10.1210/en.2007-1447 18276754

[B37] Ribet C Montastier E Valle C Bezaire V Mazzucotelli A Mairal A Viguerie N Langin D Peroxisome proliferator-activated receptor-α control of lipid and glucose metabolism in human white adipocytes Endocrinology 2010 151 1 123 133 10.1210/en.2009-0726 19887568

[B38] Schoonjans K Staels B Auwerx J Role of the peroxisome proliferator-activated receptor (PPAR) in mediating the effects of fibrates and fatty acids on gene expression J Lipid Res 1996 37 5 907 925 8725145

[B39] Tsuchida A Yamauchi T Takekawa S Hada Y Ito Y Maki T Kadowaki T Peroxisome proliferator–activated receptor (PPAR)α activation increases adiponectin receptors and reduces obesity-related inflammation in adipose tissue Diabetes 2005 54 12 3358 3370 10.2337/diabetes.54.12.3358 16306350

[B40] Mandard S Müller M Kersten S Peroxisome proliferator-activated receptor a target genes Cell Mol Life Sci 2004 61 4 393 416 10.1007/s00018-003-3216-3 14999402 PMC11138883

[B41] Krey G Braissant O L’Horset F Kalkhoven E Perroud M Parker MG Wahli W Fatty acids, eicosanoids, and hypolipidemic agents identified as ligands of peroxisome proliferator-activated receptors by coactivator-dependent receptor ligand assay Mol Endocrinol 1997 11 6 779 791 10.1210/me.11.6.779 9171241

[B42] Newman RE Bryden WL Fleck E Ashes JR Storlien LH Downing JA Dietary n-3 and n-6 fatty acids alter avian metabolism: molecular-species composition of breast-muscle phospholipids Br J Nutr 2002 88 1 19 28 12117424 10.1079/BJNBJN2002581

[B43] Randle PJ Metabolic fuel selection: general integration at the whole-body level Proc Nutr Soc 1995 54 01 317 327 10.1079/PNS19950057 7568263

[B44] Hsieh MCF Das D Sambandam N Zhang MQ Nahlé Z Regulation of the PDK4 Isozyme by the Rb-E2F1 Complex J Biol Chem 2008 283 41 27410 27417 10.1074/jbc.M802418200 18667418

[B45] Wu P Blair PV Sato J Jaskiewicz J Popov KM Harris RA Starvation increases the amount of pyruvate dehydrogenase kinase in several mammalian tissues Arch Biochem Biophys 2000 381 1 1 7 10.1006/abbi.2000.1946 11019813

[B46] Cadoudal T Distel E Durant S Fouque F Blouin J-M Collinet M Bortoli S Forest C Benelli C Pyruvate dehydrogenase kinase 4 Diabetes 2008 57 9 2272 2279 10.2337/db08-0477 18519799 PMC2518477

[B47] Connaughton S Chowdhury F Attia RR Song S Zhang Y Elam MB Cook GA Park EA Regulation of pyruvate dehydrogenase kinase isoform 4 (PDK4) gene expression by glucocorticoids and insulin Mol Cell Endocrinol 2010 315 1–2 159 167 19703515 10.1016/j.mce.2009.08.011 PMC2815206

[B48] Sibut V Hennequet-Antier C Le Bihan-Duval E Marthey S Duclos MJ Berri C Identification of differentially expressed genes in chickens differing in muscle glycogen content and meat quality BMC Genom 2011 12 112 10.1186/1471-2164-12-112 PMC3047303 21324179

[B49] Wan Z Thrush AB Legare M Frier BC Sutherland LN Williams DB Wright DC Epinephrine-mediated regulation of PDK4 mRNA in rat adipose tissue Am J Physiol Cell Physiol 2010 299 5 C1162 C1170 10.1152/ajpcell.00188.2010 20739620

[B50] Ruderman NB Saha AK Vavvas D Witters LA Malonyl-CoA, fuel sensing, and insulin resistance Am J Physiol - Endocrinol Metab 1999 276 1 E1 E18 10.1152/ajpendo.1999.276.1.E1 9886945

[B51] Gregoire F Smas C Sul H Understanding adipocyte differentiation Physiol Rev 1998 78 783 809 9674695 10.1152/physrev.1998.78.3.783

[B52] Hutley L Shurety W Newell F McGeary R Pelton N Grant J Herington A Cameron D Whitehead J Prins J Fibroblast growth factor 1 Diabetes 2004 53 12 3097 3106 10.2337/diabetes.53.12.3097 15561939

[B53] Zhu X-g Kim DW Goodson ML Privalsky ML Cheng S-Y NCoR1 regulates thyroid hormone receptor isoform-dependent adipogenesis J Mol Endocrinol 2011 46 3 233 244 10.1530/JME-10-0163 21389087 PMC3457783

[B54] Spalding KL Arner E Westermark PO Bernard S Buchholz BA Bergmann O Blomqvist L Hoffstedt J Naslund E Britton T Dynamics of fat cell turnover in humans Nature 2008 453 7196 783 787 10.1038/nature06902 18454136

[B55] Chang L Chiang SH Saltiel AR Insulin signaling and the regulation of glucose transport Mol Med 2004 10 7–12 65 71 16307172 10.2119/2005-00029.Saltiel PMC1431367

[B56] Richards MP McMurtry JP The avian proglucagon system Gen Comp Endocrinol 2009 163 1–2 39 46 18938167 10.1016/j.ygcen.2008.09.010

[B57] Richards MP McMurtry JP Expression of proglucagon and proglucagon-derived peptide hormone receptor genes in the chicken Gen Comp Endocrinol 2008 156 2 323 338 10.1016/j.ygcen.2008.01.014 18299131

[B58] Yuan JS Wang D Stewart CN Statistical methods for efficiency adjusted real-time PCR quantification Biotechnol J 2008 3 1 112 123 10.1002/biot.200700169 18074404

[B59] Bajad SU Lu WY Kimball EH Yuan J Peterson C Rabinowitz JD Separation and quantitation of water soluble cellular metabolites by hydrophilic interaction chromatography-tandem mass spectrometry J Chromatogr A 2006 1125 1 76 88 10.1016/j.chroma.2006.05.019 16759663

[B60] Waters CA Lu WY Rabinowitz JD Bassler BL Quorum sensing controls biofilm formation in Vibrio cholerae through modulation of cyclic Di-GMT levels and repression of vpsT J Bacteriol 2008 190 7 2527 2536 10.1128/JB.01756-07 18223081 PMC2293178

[B61] Benjamini Y Hochberg Y Controlling the false discovery rate: a practical and Powerful Approach to Multiple Testing J R Stat Soc Ser B 1995 57 1 289 300

[B62] Huang DW Sherman BT Lempicki RA Systematic and integrative analysis of large gene lists using DAVID bioinformatics resources Nat Protocols 2008 4 1 44 57 10.1038/nprot.2008.211 19131956

[B63] Bindea G Mlecnik B Hackl H Charoentong P Tosolini M Kirilovsky A Fridman WH Pages F Trajanoski Z Galon J ClueGO: a Cytoscape plug-in to decipher functionally grouped gene ontology and pathway annotation networks Bioinformatics 2009 25 8 1091 1093 10.1093/bioinformatics/btp101 19237447 PMC2666812

